# Emergence of the Stoner-Wohlfarth astroid in thin films at dynamic regime

**DOI:** 10.1038/s41598-017-13854-7

**Published:** 2017-10-18

**Authors:** José Luis F. Cuñado, Alberto Bollero, Tomás Pérez-Castañeda, Paolo Perna, Fernando Ajejas, Javier Pedrosa, Adrian Gudín, Ana Maldonado, Miguel Angel Niño, Rubén Guerrero, David Cabrera, Francisco J. Terán, Rodolfo Miranda, Julio Camarero

**Affiliations:** 1 Departamento de Física de la Materia Condensada and Instituto “Nicolás Cabrera”, Universidad Autónoma de Madrid, Madrid, Spain; 2 Instituto Madrileño de Estudios Avanzados en Nanociencia IMDEA-Nanociencia, Campus Universidad Autónoma de Madrid, Madrid, Spain

## Abstract

The Stoner-Wohlfarth (SW) model is the simplest model that describes adequately the magnetization reversal of nanoscale systems that are small enough to contain single magnetic domains. However for larger sizes where multi-domain effects are present, e.g., in thin films, this simple macrospin approximation fails and the experimental critical curve, referred as SW astroid, is far from its predictions. Here we show that this discrepancy could vanish also in extended system. We present a detailed angular-dependent study of magnetization reversal dynamics of a thin film with well-defined uniaxial magnetic anisotropy, performed over 9 decades of applied field sweep rate (d*H*/d*t*). The angular-dependent properties display a gradual transition from domain wall pinning and motion-like behaviour to a nucleative single-particle one, as d*H*/d*t* increases. Remarkably, in the high dynamic regime, where nucleation of reversed domains is the dominant mechanism of the magnetization reversal (nucleative regime), the magnetic properties including the astroid become closer to the ones predicted by SW model. The results also show why the SW model can successfully describe other extended systems that present nucleative regime, even in quasi-static conditions.

## Introduction

The study of magnetization reversal processes is an important issue in Nanomagnetism today^1^, both from fundamental and technological points of view. The use of magnetic materials in all applications ranging from compass needles to electrical motors, cellular phones and personal computers has triggered the search for materials with particular properties concerning both their static and dynamic reversal behavior. For instance, magnetization reversal features (i.e., relevant mechanism, reversal times, reversal fields) determine how the magnetization preserves its state in powerful permanent magnet applications, based on nanograin ferromagnetic structures consolidated in bulk systems^2^, as well as how the information can be read and written in spintronic devices^3^, based on multilayered thin film architectures. Current and future technologies require hence basic understanding and control of hysteresis and magnetization reversal processes^4^.

**Figure 1:**
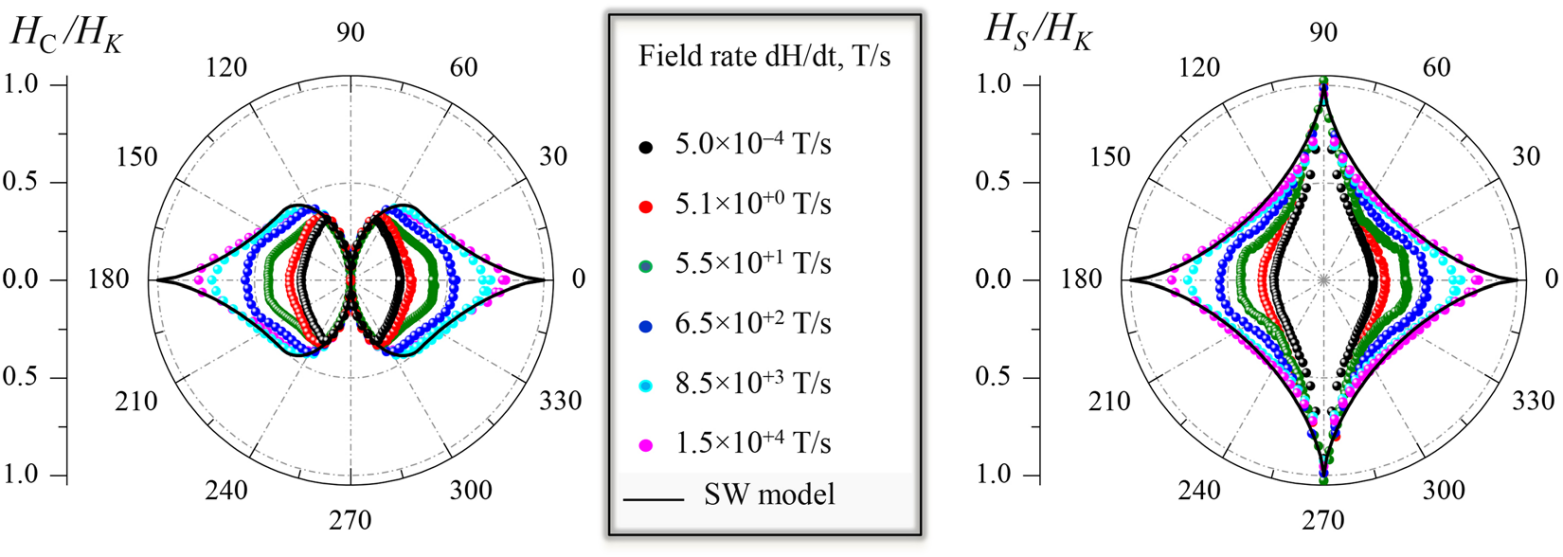
*Emerging single-particle like behavior in thin films*. Angular dependence of the dynamic coercivity ${\mu }_{{\rm{0}}}{H}_{{\rm{C}}}$ (left polar plot) and switching field ${\mu }_{{\rm{0}}}{H}_{{\rm{S}}}$ (right polar plot) for a thin film with well-defined uniaxial anisotropy. Symbols are the experimental data normalized to the anisotropy field ${\mu }_{{\rm{0}}}{H}_{{\rm{K}}}$ derived from vectorial-resolved kerr hysteresis loops acquired at the indicated applied field sweep rate (d*H*/d*t*) values. Solid lines are the expected values from the Stoner-Wohlfarth (SW) model that assumes uniform rotation and switching of the entire system. Notice that the experimental data are approaching to the SW prediction as d*H*/d*t* increases.

The physical mechanisms responsible for the hysteresis and magnetization reversal in magnetic nanostructures become more complex to interpret as the dimensionality increases^5–7^, from zero-dimensional (nanoparticles) to two-dimensional (thin films). In general, hysteresis include reversible (rotation) and irreversible (switching) magnetic transitions. Magnetization reversal can take place in different ways, depending on object size and physical parameters. The exchange interaction, magnetic anisotropy and magnetostatics are among the most important physical parameters involved^8^. First, exchange interaction favors uniform magnetization configurations. Second, magnetic anisotropy favors the orientation of the magnetization vector along certain preferred directions. And third, magnetostatic interaction favors configurations giving null average magnetic moment, in contrast with anisotropy and exchange. In addition to these intrinsic parameters, the hysteresis depends on extrinsic parameters such as temperature, applied field angle, and applied field sweep rate. Models for collective reversal provide good agreement between experiment and theory in the case of small particles^9^, both in uniform reversal modes (SW model^5^), as shown for nanoparticles^10^, or in non-uniform ones, as in nanowires^11^. The geometric representation of SW model is recognized by the polar plot representation (as a function of the applied field angle) of the reversal fields, referred to both the coercivity and the (irreversible) switching field, called SW-astroid^12^, which are represented with solid lines in the left and right panel of Fig. 1, respectively. What stands out the most is that the SW model predicts identical switching fields at the easy-axis (e.a.) and hard-axis (h.a.) magnetization directions, whereas the curling model predicts smaller values at e.a. direction^13^, depending on the aspect ratio of the one-dimensional magnetic nanostructure. For microscopic objects and extended thin films, reversible and irreversible magnetization transitions represent different reversal processes, and they are often a manifestation of the magnetic symmetry of the system^14^. Reversible transitions are related to rotation processes, whereas irreversible ones are related to nucleation of reversed magnetic domains (usually created at inhomogeneities or defects) and subsequent propagation of their magnetic domain walls, under the field pressure^15^.

The magnetic properties are hence determined by a combination of nucleation and domain wall propagation behaviors with their associated energy barriers. In addition, the relevance of these processes depends on the direction of the applied magnetic field with respect to certain characteristic axes of the magnetic system, i.e., magnetic anisotropy axes^16^, and depends as well on the field rate of change, since they are different in quasi-static and dynamic regimes^17^. In magnetic thin films with in-plane dimensions considerably larger than the domain wall width the irreversible processes usually starts at magnetic fields that are significantly lower than expected from SW model, the so-called Brown’s paradox^18^. In this case, the lateral dimensions strongly affect the magnetic behavior. For instance, experimental astroids with shapes approaching those predicted by SW model have been found in $200\times 500$ nm^2^ size thin film-based magnetic elements, but become much less ideal when the size increases, even though still in the submicron size^19^. In this sense, predictions based on the SW model are significantly far from experimental data for extended thin film-based magnetic nanostructures.

In this letter we show the emergence of a single-particle like magnetic behavior in extended thin films at dynamic regime. This can be easily visualized in Fig. 1 by observing that the dynamic coercive field (${\mu }_{{\rm{0}}}{H}_{{\rm{C}}}$) and switching field (${\mu }_{{\rm{0}}}{H}_{{\rm{S}}}$) are approaching to the SW prediction as the applied field sweep rate (d*H*/d*t*) increases. We have carried out a detailed dynamical study on the angular dependence of the magnetic properties on model extended thin films with well-defined uniaxial magnetic anisotropy. In particular, we have used Co FM thin films grown on Si substrates with Ta buffer layer. Samples were exposed to 0.2 Tesla in-plane external magnetic field during growth, in order to induce a well-defined uniaxial magnetic anisotropy (${K}_{{\rm{U}}}$) in the FM layer, parallel to the field direction. Experimental details are given in Methods and in the Supplementary information. The magnetization reversal dynamics of the two in-plane magnetization components, i.e., parallel (${M}_{||}$) and transverse (${M}_{\perp }$) to the external applied field, have been investigated over 9 decades of d*H*/d*t* and in the whole angular range. Thermal activated dynamical effects are found during irreversible processes and depend strongly on the orientation of the anisotropy axis with respect to the external field (referred as the angle ${\alpha }_{{\rm{H}}}$). The effects are more relevant near the e.a. direction (${\alpha }_{{\rm{H}}}{\mathrm{=0}}^{\circ }$) and vanishes near the h.a. direction (${\alpha }_{{\rm{H}}}{\mathrm{=90}}^{\circ }$). In contrast, the reversible transitions are not affected by dynamical effects. The data have been interpreted in the framework of the magnetic domain pinning and rotation models for quasi-static and dynamic conditions, respectively.

**Figure 2:**
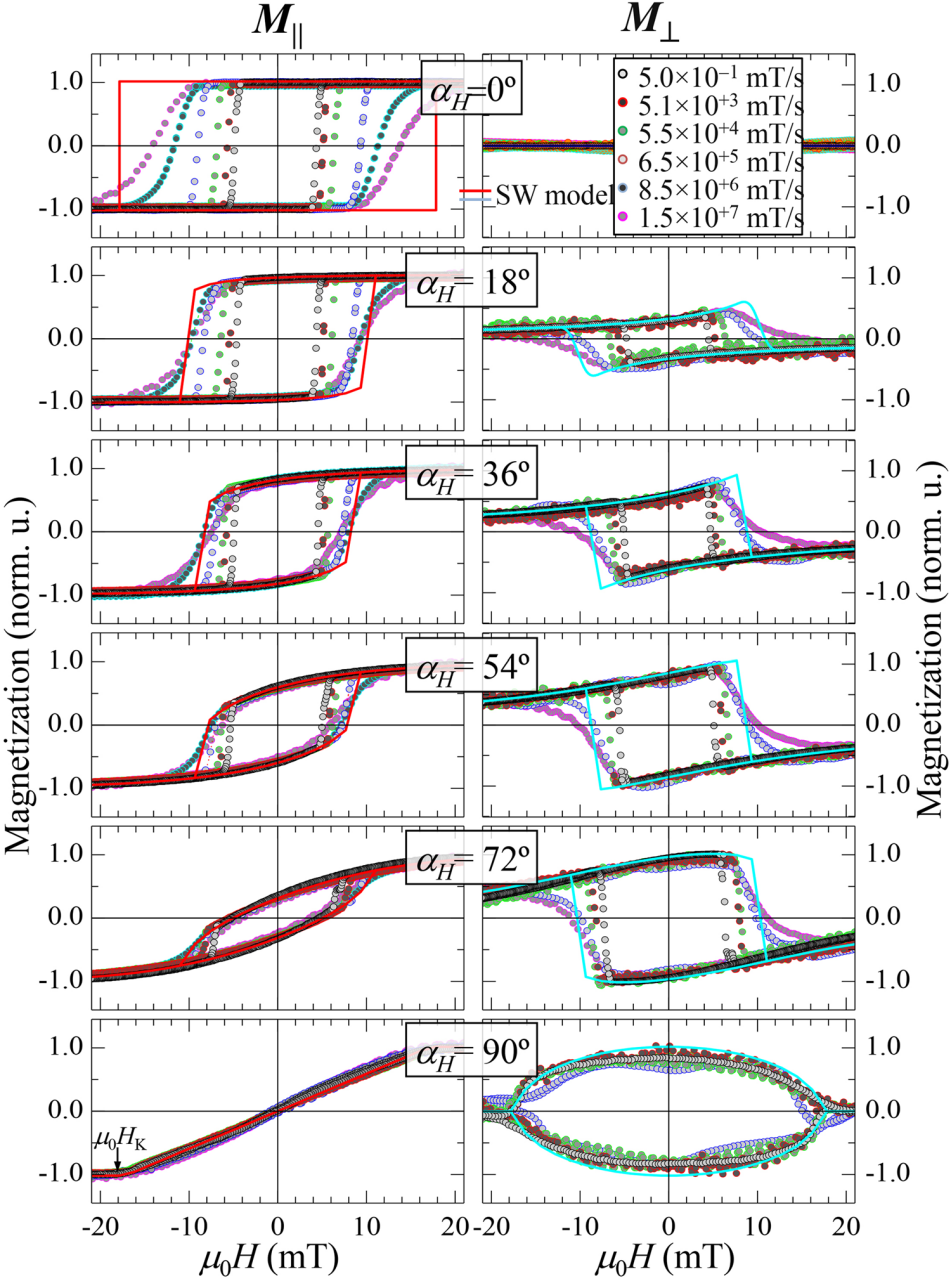
*Angular-dependent dynamical hysteresis loops*. Angular-dependent dynamical hysteresis loops for a FM thin film with well-defined uniaxial magnetic anisotropy. The applied magnetic field angle ${\alpha }_{{\rm{H}}}$ and sweep rate d*H*/d*t* are indicated. For each ${\alpha }_{{\rm{H}}}$, both parallel ${M}_{||}(H)$ (left plots) and transversal ${M}_{\perp }(H)$ (right plots) magnetization Kerr hysteresis loops were acquired simultaneously at room temperature for several d*H*/d*t* values. The solid lines correspond to the predicted ones from SW model using the experimental anisotropy field ${\mu }_{{\rm{0}}}{H}_{{\rm{K}}}$ indicated in the bottom graph.

The reversal processes can be determined directly by a simple inspection of the in-plane vectorial magnetization loops^20,21^, highlighting the importance of vectorial-resolved magnetometry. Full angular-dependent studies were carried out at selected d*H*/d*t* values. A detailed analysis in quasi-static condition is given in the Supplementary information. Figure 2 shows representative parallel (${M}_{||}$) and transversal (${M}_{\perp }$) magnetization loops at selected ${\alpha }_{{\rm{H}}}$ acquired at different d*H*/d*t*. For a given $dH/dt$, in general, irreversible (sharp) transitions and/or fully reversible (smoother) transitions are observed in both ${M}_{||}(H)$ and ${M}_{\perp }(H)$ loops, which correspond to nucleation of magnetic domains followed by domain wall propagation and to rotation processes, respectively. The relative weight of these two reversal mechanisms depends on ${\alpha }_{{\rm{H}}}$ and $dH/dt$ and reveals the uniaxial magnetic anisotropy of the film.

For a given ${\alpha }_{{\rm{H}}}$, the shapes that both ${M}_{||}(H)$ and ${M}_{\perp }(H)$ loops have before the irreversible transition are similar for the whole dynamic range of d*H*/d*t* investigated, as can be seen on the left and right graphs of Fig. 2, respectively: dynamical effects result in a lengthening of the reversible processes, but without changing their shape, which indicates that reversible processes are non-thermally activated. Dynamical effects are only observed in the irreversible transitions: increment of the reversal fields and broadening of the transitions as the applied field sweep rate is increased. As for the first aspect, the reversal field enhancement indicates that the irreversible processes are thermally activated. For the second aspect, the broadening of the irreversible transitions suggests that the reversal is governed by two different mechanisms depending on the dynamic regime: at lower d*H*/d*t* the reversal is mainly governed by domain wall propagation processes (characterized by more abrupt jumps), while at higher sweep rates, domain nucleation processes dominate (softening the irreversible jumps), as discussed in more detail below.

Over the last decades, several studies have addressed the magnetization reversal dynamics in several magnetic thin film-based nanostructures at different timescales. In general, precessional reversal of magnetization takes place at subnanosecond timescales (ultra-fast dynamic regime)^22^, while nucleative and propagative processes can take variable amounts of time^7^, from submicrosecond (high dynamic regime) to large fractions of a second (quasi-static or low dynamic regime)^23^. For instance, hysteresis loop measurements as a function of the applied magnetic field sweep rate (d*H*/d*t*) support the picture of nucleation-dominated reversal in the high dynamic regime and wall propagation-dominated reversal in the low dynamic regime^24,25^. This scenario has been found in dynamical studies of single ferromagnetic (FM) layers with both in-plane^26,27^ and out-of-plane^28^ anisotropy as well as in more complex structures, such as in-plane^29,30^ and out-of-plane^31^ exchange-biased FM/antiferromagnetic systems and trilayer FM1/non magnetic/FM2 magnetic structures^32^. The crossover between propagative and nucleative regimes depends on the strength of the anisotropy of the system: higher anisotropy favors earlier nucleative regimes. It is worth mentioning that all these studies were performed only at the e.a. direction, and with sensitivity only to the magnetization component parallel to the external field.

**Figure 3:**
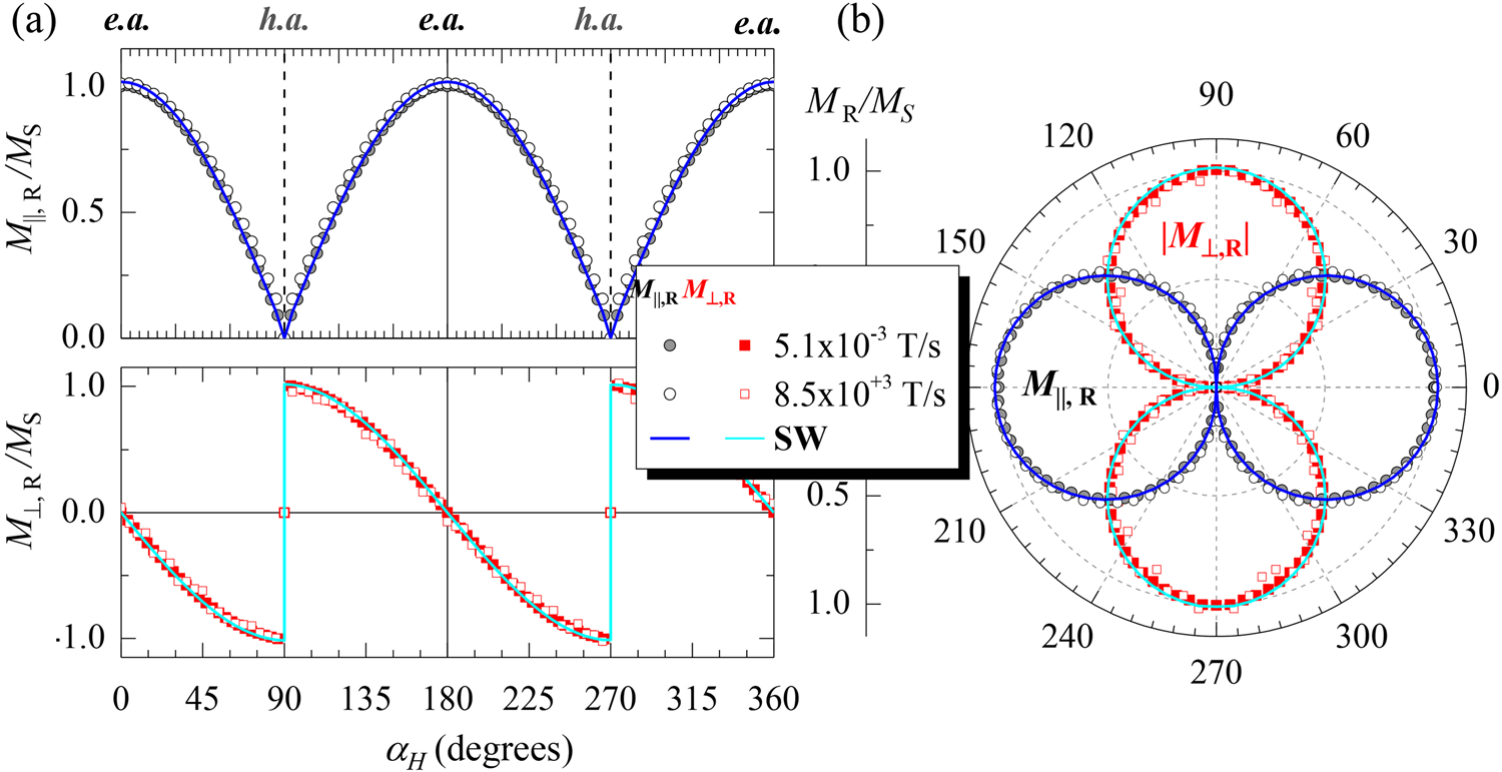
Dynamic remanence and magnetic symmetry. (**a**) Angular dependence of the normalized remanent magnetization components of a well-defined uniaxial magnetic anisotropy system at indicated d*H*/d*t* values. For clarity, ${M}_{||,R}$ and ${M}_{\perp ,R}$ are depicted in separated plots, top and bottom respectively. (**b**) Corresponding polar plot representation. Symbols represent the experimental data extracted from the in-plane resolved hysteresis loops acquired in quasi-static (circles) and at high dynamic (squares) conditions, as the ones shown in Fig. 2. The solid lines correspond to theoretical evolution derived from the SW model.

In our study, in addition, we can determine the angular dependence of the dynamical effects. For instance, by comparing the loops of Fig. 2 acquired at the minimum and maximum d*H*/d*t* values for the different angles, we find out that the rise of the dynamic effects are more pronounced at the e.a. direction (top graph), i.e., ${\alpha }_{{\rm{H}}}{=0}^{\circ }$, diminishing progressively as the applied field angle increases (middle graphs), and vanishing at the h.a. direction (bottom graph), i.e., ${\alpha }_{{\rm{H}}}{=90}^{\circ }$. Notice that as d*H*/d*t* increases, the experimental loops approaches the ones predicted with the SW model (solid lines in Fig. 2). In turn, the remanent magnetization (or remanence) ${M}_{{\rm{R}}}$, i.e., magnetization at zero field, of both magnetization components is independent of the applied field sweep rate in the whole angular range. Figure 3 shows similar angular dependence at two different d*H*/d*t* values that differ in six orders of magnitude, i.e., for quasi-static and high dynamic conditions. The corresponding polar plots depicted in Fig. 2(b) show the characteristic (two-fold) symmetry of a well-defined uniaxial magnetic anisotropy system: ${M}_{{\rm{R}},\parallel }({\alpha }_{{\rm{H}}})$ and $|{M}_{\perp ,{\rm{R}}}({\alpha }_{{\rm{H}}})|$ display *“two-lobes”* shape rotated 90° with respect to each other. This unambiguously indicates that the orientation of the magnetization vector at remanence does not present dynamical effects. In fact, this is preserved until the irreversible transition takes place. These features are in accordance with the relevance of the (non-thermal activated) reversible and (thermal activated) irreversible transitions. Therefore, the magnetic symmetry is preserved and larger dynamic effects are found where irreversible processes are more relevant during reversal.

**Figure 4:**
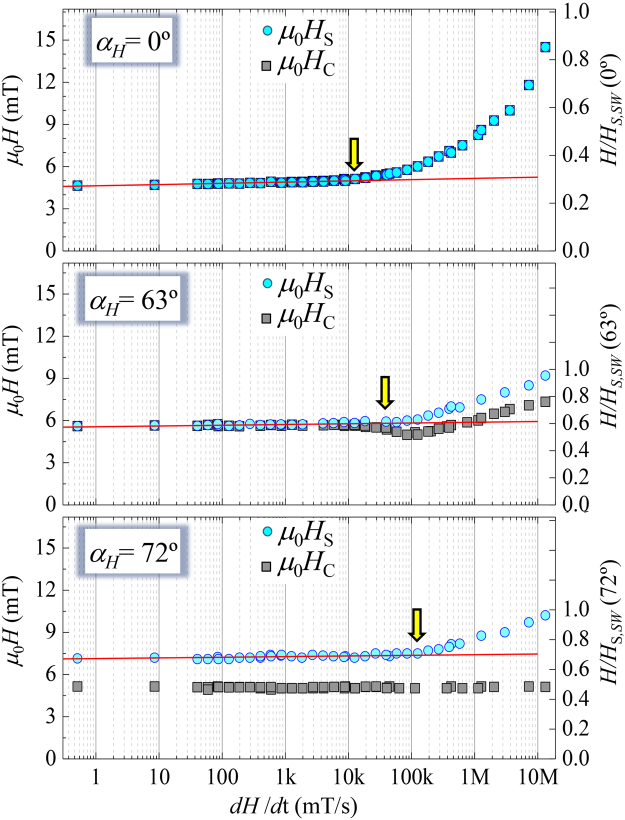
*Dynamic coercivity and switching field*. Evolution of the coercive field ${\mu }_{{\rm{0}}}{H}_{{\rm{C}}}$ (square symbols) and switching field ${\mu }_{{\rm{0}}}{H}_{{\rm{S}}}$ (circles) as a function of the applied field sweep rate d*H*/d*t* for selected applied field angles ${\alpha }_{{\rm{H}}}$. The right-Y axes have been normalized by the corresponding switching fields predicted by the SW model (${H}_{S,\mathrm{SW}}({\alpha }_{{\rm{H}}})$). The symbols are the experimental data taken from the in-plane resolved magnetization curves such as those shown in Fig. 2. The lines are the expected behavior predicted by a phenomenological model based on domain wall dynamics (see text). A semi-logarithmic scale has been used to identify the range of sweep rates where domain wall propagation dominates the reversal^29,30^. Vertical arrows remark the crossover between the propagative to nucleative regime.

Thermal *vs*. non-thermal activation effects on magnetization reversal dynamics manifest directly through the analysis of the dynamic transition reversal fields of both magnetization components, referred as coercive field ${\mu }_{{\rm{0}}}{H}_{{\rm{C}}}$ and switching field ${\mu }_{{\rm{0}}}{H}_{{\rm{S}}}$, and defined as the fields at which ${M}_{||}({\mu }_{{\rm{0}}}{H}_{{\rm{C}}}\mathrm{)=0}$ and ${M}_{\perp }({\mu }_{{\rm{0}}}{H}_{{\rm{S}}}\mathrm{)=0}$, respectively. Figure 4 shows the corresponding dynamic evolution of coercive and switching fields at representative ${\alpha }_{{\rm{H}}}$. The global behavior is summarized as an increase in ${\mu }_{{\rm{0}}}{H}_{{\rm{S}}}$ as d*H*/d*t* increases, but clearly two dynamical behaviors at low and high sweep rates can be distinguished, where ${\mu }_{{\rm{0}}}{H}_{{\rm{S}}}$ varies very slowly at low sweep rate, and increases more rapidly at high rates. The crossover between the two regimes, marked with vertical arrows in Fig. 4, takes place at higher d*H*/d*t* for larger angles (yellow arrow shifts to the right as angle is increased). In contrast, ${\mu }_{{\rm{0}}}{H}_{{\rm{C}}}$ shows three dynamical evolutions for different angular ranges. In general, all can be associated to the relevant mechanism during reversal, where (thermally activated) irreversible and (non-thermally activated) reversible processes govern the dynamics around the e.a. and h.a. directions, respectively. For intermediate angles, the dynamic fields result from the competition between them.

Around the e.a. direction, for ${\alpha }_{{\rm{H}}}\, < \,{45}^{\circ }$, both reversal fields are taken just when the reversible transition have finished and similar dynamic coercive and switching field values are found, which depend on both d*H*/d*t* and ${\alpha }_{{\rm{H}}}$ (see top graph of Fig. 4). In general, the reversal fields increase slowly and logarithmically in d*H*/d*t* for low sweep rates (low dynamic regime). For high sweep rates the increase of the reversal field with field sweep rate becomes much faster (high dynamic regime). This behavior has been explained in the literature in terms of a transition between two different thermally activated reversal regimes^28–30^. At low sweep rates the magnetization reverses mainly by domain wall propagation while at higher sweep rates, where the propagation process becomes relatively slow compared to the variation of the applied magnetic field, successive nucleations of reversed domains dominate the reversal. Since the activation energy for domain nucleation is larger than that for domain wall motion, the reversal field varies more strongly with d*H*/d*t* in this reversal regime. In addition, in both regimes the dynamic reversal field increases and the rising slope decreases as ${\alpha }_{{\rm{H}}}$, increases. As a result, the crossover between these two thermally activated regimes is found at higher d*H*/d*t* as we move away the e.a. direction.

For ${\alpha }_{{\rm{H}}}\, > \,{45}^{\circ }$, while the dynamic switching field shows the trend already described, the dynamic coercivity presents a peculiar evolution in which three behaviors for different d*H*/d*t* ranges can be distinguished, defining a low, crossover and high dynamical regime regions, respectively (see central graph of Fig. 4). Two of these regions (low and high dynamic regimes) present an approximately linear relationship between ${\mu }_{{\rm{0}}}{H}_{{\rm{C}}}$ and $log$(d*H*/d*t*) but with different slope, which is higher at high dynamic regime. A third region characterized by a dip in ${\mu }_{0}{H}_{{\rm{C}}}$ is found at the crossover from low dynamic regime to high dynamic regime. Similar behavior was found in epitaxial Fe/GaAs(001) films, where the dip was shifted to lower d*H*/d*t* as the temperature was reduced^33^. It has been suggested that forced depinning from nucleation sites at these sweep rates is responsible for the dip in coercive field, and that there are two distinct timescales associated with the reversal process which correspond to domain nucleation and wall motion, respectively. In our case, this behavior can be simply understood taking into account that for ${\alpha }_{{\rm{H}}}\, > \,{45}^{\circ }$ the dynamic coercive field can be derived from a still reversible process, happening before the irreversible transition related to the switching field takes place. Therefore, for this angular range, nucleation dynamics cannot be derived from the dynamic coercivity, but from the dynamic switching field. In fact, the latter presents two clear dynamical behaviors following the trend discussed above, i.e., propagative to nucleative regimes. In addition, the crossover between the two regimes still shifts to higher d*H*/d*t* values when ${\alpha }_{{\rm{H}}}$ increases.

Around the h.a. direction, different dynamic ${\mu }_{{\rm{0}}}{H}_{{\rm{C}}}$ and ${\mu }_{{\rm{0}}}{H}_{{\rm{S}}}$ values are found, as expected from the definition of both reversal fields, which depend on ${\alpha }_{{\rm{H}}}$. In this angular range the coercivities are taken long before the reversible processes have finished, resulting in a non-dependency of ${\mu }_{{\rm{0}}}{H}_{{\rm{C}}}$ with d*H*/d*t*, and in a decrease as ${\alpha }_{{\rm{H}}}$ increases, vanishing when approaching the h.a. direction. In turn, ${\mu }_{{\rm{0}}}{H}_{{\rm{S}}}$ increases as ${\alpha }_{{\rm{H}}}$ and d*H*/d*t* increase. At low dynamic regime the switching field increases, with a maximum at the h.a., and its dynamic slope decreases approaching the h.a. direction. This indicates that the magnetization reversal dynamics close to the h.a. is governed mainly by non-thermally activated rotation processes.

The low dynamic regime of the irreversible transition can be simulated by using a phenomenological model based on thermally activated relaxation “single relaxation time approximation” of propagating magnetic domains walls^24,25^. The model assumes that the energy barrier for magnetization reversal varies linearly with the applied magnetic field (i.e., domain wall propagation with weak pinning centers) and predicts a logarithmic dependence of the reversal field on the applied sweep rate (equation (7) of^28^). We want to point out that this analysis has been performed in ${\mu }_{0}{H}_{{\rm{S}}}$(d*H*/d*t*) because the switching field is sensitive just to irreversible processes for the whole angular range. Notice that, although this is also valid for the dynamic coercivity for ${\alpha }_{{\rm{H}}} < {45}^{\circ }$, it is not correct for larger angles where mixed reversible and irreversible processes are present. The solid lines of Fig. 4 derived from this model reproduce the experimental dynamic evolution for the low dynamic regime. Moreover, the d*H*/d*t* validity range of the simulation indicates that the transition from the domain wall propagation regime at lower d*H*/d*t* to the domain nucleation regime at higher d*H*/d*t* shifts to higher sweep rates as ${\alpha }_{{\rm{H}}}$ increases (see arrows in Fig. 4). This indicates that the higher irreversible field values found as we move away from the e.a. make nucleation processes more relevant and delay the transition between the dynamic regimes.

**Figure 5:**
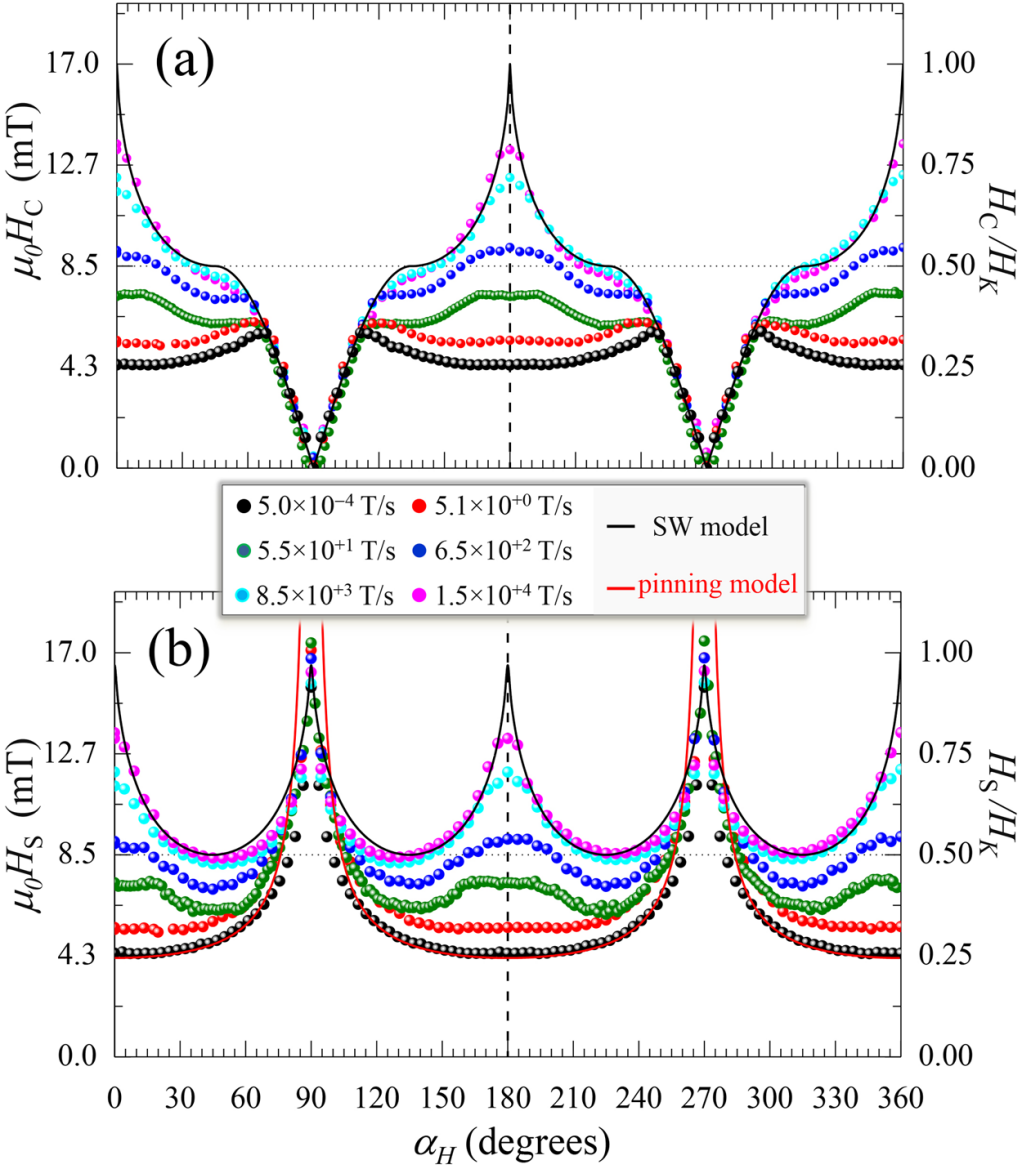
*Angular dependence of dynamic field transitions*. Angle-dependent coercive field ${\mu }_{{\rm{0}}}{H}_{{\rm{C}}}$ (**a**) and switching field ${\mu }_{{\rm{0}}}{H}_{{\rm{S}}}$ (**b**) for selected applied magnetic field sweep rates d*H*/d*t*. The symbols are obtained from the experimental vectorial-resolved dynamic hysteresis curves (see for instance cycles in Fig. 2), as described in the text. The polar plot representation of this data is shown in Fig. 1. The direct comparison between experiment and the predictions derived from the pinning model (red line) and the SW model (black lines) indicates a gradual transition between pinning-like behavior and SW-like one as d*H*/d*t* increases.

The tendency in the high dynamic regime, i.e., where nucleation becomes more relevant during the irreversible transition, can be figured out by simply inspecting the angular dependence evolution of the coercive and switching fields as d*H*/d*t* increases. Figure 5 compares the data extracted from the experimental loops (symbols) with the predicted ones by the domain wall pinned (dashed line) model and the Stoner-Wohlfarth (SW) (solid line) models. It is worth noting that the experimental data show a clear transition from a pinned-like behavior at low d*H*/d*t* (propagative regime) to a SW-like behavior at high d*H*/d*t* (nucleative regime).

The SW model assumes a single particle behavior, i.e., coherent reversal by switching (irreversible process) and/or rotation (reversible) of the whole magnetization, without taking into account any other irreversible magnetization reversal process like nucleation and propagation of magnetic domains. The latter is, in fact, energetically much more favorable in extended systems, in which defects of any kind–structural or morphological–play the main role to activate irreversible magnetic domain nucleation processes at magnetic fields that are significantly lower than expected by the SW model. In fact, low coordination sites such as sample edges, grain boundaries, and topographic roughness, are unavoidable in real samples. These defects can act as pinning centers for the created magnetic domain walls and the reversal subsequently continues via domain wall propagation. In this case, a simple model of pinned 180° magnetic domain walls (pinning model)^34^ predicts a $\mathrm{1/|}cos{\alpha }_{{\rm{H}}}|$ law for the angular dependence of the reversal field, i.e., only the projection of the field along the magnetization is effective. As a consequence coercive field is no longer maximum at the e.a. direction, in clear contrast with the SW model that predicts a maximum value of the coercive field, which is similar to the anisotropy field.

From our dynamic study, at low d*H*/d*t* the angular evolution of the irreversible transition shows a pinned-like behavior in a broad angular range around the e.a. direction, In particular, both ${H}_{{\rm{C}}}({\alpha }_{{\rm{H}}})$ and ${H}_{{\rm{C}}}({\alpha }_{{\rm{H}}})$ follow the predicted behavior by the pinning model for an angle range $\pm {60}^{\circ }$ (see red solid line in Fig. 5(b)). Similar findings have been reported in both perpendicular^35^ and in-plane^36^ anisotropy systems, from angular-dependence quasi-static measurements. The experimental data increase as d*H*/d*t* increases, but always limited by the expected values from the SW model (see black solid lines in Fig. 5). Remarkably, the similarity between the experimental data at the highest dynamic regime investigated and those predicted by the SW model are very high. Dynamic measurements performed just at the e.a. direction in perpendicular anisotropy systems also showed this tendency, i.e., the coercive field at the e.a. direction becomes closer to the anisotropy field at the fastest dynamical regime^37^. In addition, we show that the whole angular range of transition fields, including both coercive and switching fields, approaches the SW-like behavior as d*H*/d*t* increases (Fig. 5). The transition from the pinned to the SW model with increasing d*H*/d*t* can be observed clearly in the polar representation shown in Fig. 1. Thus, our dynamic study shows that pinned-like and SW-like behaviors can be found at low d*H*/d*t* and high d*H*/d*t*, respectively. Moreover, the data show that this is directly connected with propagative and nucleative regimes, respectively.

The results show that the dynamic properties, including hysteresis, remanences and reversal fields, approach the SW predictions when nucleation processes dominate the magnetization reversal, leading to provide the general trend of the dynamic magnetic properties of other extended magnetic systems. In general, during the irreversible transition the magnetization reverses by nucleation of domains (oriented parallel to the anisotropy axis) and propagation of domain walls. The nucleation sites are associated with defects (or local weakening of the intrinsic anisotropy) and the domain wall propagation is hindered by local pinning centers, as described above. The ratio between the rates of domain wall propagation and nucleation events is highly sensitive to both applied field angle and the applied field sweep rate. Respect to the later, the irreversible transitions become wider as dH/dt increases indicating the presence of a distribution of nucleation energy barriers. In the low dynamic regime, before the crossover, the lowest-energy nucleation barriers are overcome and the domain walls expand driven by the external magnetic field. At high dynamic regime, above the crossover, nucleative processes dominate and both coercivity and switching fields approach the SW predictions as d*H*/d*t* increases. At the fastest d*H*/d*t*, the whole extended system behaves as a single-like particle because high nucleation rates can inhibit propagative events. Within this general scenario, the crossover between the propagative to nucleative regime would depend strongly on intrinsic properties of the magnetic system, as mentioned below.

### Summary

We have demonstrated not only that a single-particle like magnetic behavior can be found in extended magnetic systems but also for what dynamic conditions, i.e., when nucleative processes govern the reversal. The detailed vectorial-resolved angular-dependent study of a model system, performed over 9 decades of d*H*/d*t* and in the whole angular range, provides a general view on the dynamics of magnetization reversal processes. In general, the data show that while thermal activation processes take place during the irreversible transitions, which correspond to nucleation and propagation of magnetic domains, reversible transition via rotation processes are not thermally activated. Propagative and nucleative processes govern the reversal at low d*H*/d*t* and high d*H*/d*t*, respectively. The transition between both regimes depends on ${\alpha }_{{\rm{H}}}$, increasing as ${\alpha }_{{\rm{H}}}$ increases. Dynamical effects are also shown on the angular dependence of the transition fields, where a transition from pinned-like to SW-like behaviors has been found. In particular, the reversal fields behave accordingly to the pinning model for the propagative regime whereas they become closer to the SW model for the nucleative regime, i.e., emerging the Stoner-Wolfhart astroid at dynamic regime as Fig. 1 illustrates.

This study also demonstrates that nucleation processes governing magnetization reversal is at the heart of coercivity enhancement, which is the path for the development of high performance permanent magnets^38^, as well as it explains why the SW model can describe successfully the angular-dependence properties of more complex extended systems, i.e., exchange biased ferromagnetic/antiferromagnetic bilayers^39–42^ and ripple patterned films^43^, where nucleative processes are much more relevant even in quasi-static conditions. This study emphasizes the importance of dynamical effects in order to understand and control magnetic systems.

## Methods

Detailed description on sample preparation and experimental are described in the Supplementary information. The data presented here correspond to a 20 nm thick Co thin film with well-defined uniaxial magnetic anisotropy. Angular-dependent, time-resolved, vectorial-resolved Kerr magnetometry measurements were performed at room temperature over 9 decades of applied magnetic field sweep rates. With this magnetometry, magnetization reversal dynamics of the two in-plane magnetization components, i.e., parallel (${M}_{||}$) and transverse (${M}_{\perp }$) to the external applied field has been explored.

The reliability of the dynamical study presented here rests on the accurate dynamic characterization of the time-resolved v-MOKE setup developed, focusing on both electromagnet and detection system. A non-desirable dynamic magnetic response of the electromagnet (i.e., vanishing the linear relationship between the applied current and the induced magnetic field, with opening of the current-field hysteresis loop of the electromagnet) and/or non-timely response (artificial time delay) of the detection system (either from the twin photodiode detector system or the digital oscilloscope) would promote artefacts in the dynamic magnetic measurements. These would display (artificial) magnetic hysteresis loop opening, independently of the applied field angle. The loop opening would provide (wrong) larger reversal fields and remanence values, being the measure more erroneous the higher the applied field sweep rate. In order to avoid this, we have checked that the magnetic field response of the electromagnet is linear with the driving current in the whole dynamic range investigated, i.e., without loop opening, and the rise-time of the detector system has been set to nanosecond timescale. A cross-check, that ensures that both electromagnet and detection system have been properly chosen, comes from the experimental observations of similar dynamic angular-dependent remanence values (see Fig. 3) and non-hysteretic loops at the hard-axis direction in the whole dynamical range investigated (see bottom left graph of Fig. 2).

## Electronic supplementary material

Supplementary information
