# Supplementary material for: Emergence of the Stoner-Wohlfarth astroid in thin films at dynamic regime
Source: Sci Rep. 2017 Oct 18;7:13474. doi: 10.1038/s41598-017-13854-7 (PMC5647445; doi:10.1038/s41598-017-13854-7)
Supplement: Supplementary file 1 — Supplementary information [file 41598_2017_13854_MOESM1_ESM.pdf]

Supplementary information:

Emergence of the Stoner-Wolfhart astroid in thin films at dynamic regime

José Luis F. Cuñado,[1,2] Alberto Bollero,[2] Tomás Pérez-Castañeda,[1] Paolo Perna,[2] Fernando Ajejas,[1] Javier Pedrosa,[2] Adrian Gudín,[2] Ana Maldonado,[1] Miguel Angel Niño,[2] Rubén Guerrero,[2] David Cabrera,[2] Fran J. Teran,[2] Rodolfo Miranda,[1,2] and Julio Camarero[1,2,*]

[1]Departamento de Física de la Materia Condensada and Instituto "Nicolás Cabrera", Universidad Autónoma de Madrid, 28049 Madrid, Spain

[2]Instituto Madrileño de Estudios Avanzados en Nanociencia IMDEA-Nanociencia, Campus Universidad Autónoma de Madrid, 28049 Madrid, Spain

We described the experimental details of the angular-resolved dynamical magnetic study presented in the main body of the manuscript, performed in model thin films with well-defined uniaxial magnetic anisotropy. A detailed analysis of the data acquired in quasi-static conditions is also presented. The data are discussed in the framework of both domain wall pinning and macrospin Stoner-Wohlfarth reversal models.

EXPERIMENTAL

A schematic representation of the sample structure and the experimental configuration are shown in Fig. S1. Co thin films were fabricated by DC magnetron sputtering at room temperature (RT) on thermally oxidized Si substrates covered with a 2 nm thick Ta buffer layer to avoid oxidation from the substrate. The samples were grown in a 0.2 T in-plane external magnetic field to induce a well-defined uniaxial magnetic anisotropy (K_{U}) in the ferromagnetic (FM) layer, with the anisotropy direction parallel to the field direction. The samples were finally capped with a 2 nm thick Ta layer to prevent oxidation. Three samples with different thicknesses were prepared, $t_{\mathrm{Co}} = 5$, 10, and 20 nm, all of them presenting the aforementioned uniaxial magnetic anisotropy. Similar quasi-static and dynamic behaviors have been found for the three samples. Here we present a detailed analysis of the quasi-static properties of the thicker FM layer whereas the dynamical aspects are discussed in the manuscript. Quasi-static conditions in this context represents a field sweep rate in the order of a few mT/s. The frontier between what is called quasi-static and dynamic regimes will be more accurately stablished in the main body.

Angular dependent, time-resolved, vectorial Kerr magnetometry measurements have been performed with our home-made vectorial magneto-optic Kerr effect (v-MOKE) setup at RT over 9 decades of applied magnetic field sweep rates ($\mathrm{d}H/\mathrm{d}t$). The basics of the experimental setup has been described elsewhere [1]. In this case, detectors and electromagnets have been adapted to suit the requirements of dynamical measurements over a wide range of $\mathrm{d}H/\mathrm{d}t$, from 10^{-4} to 10^{+4} T/s. In particular, the magnetic field response of the electromagnet is linear with the driving current up to 80 mT for frequencies ranging from 1 mHz to 150 kHz, and the rise-time of the detectors has been set to 20 ns. A cross-check that ensures that both electromagnet and detection systems are properly chosen comes from the experimental observation of non-hysteretic loops at the hard-axis direction in the whole dynamical range investigated (see bottom left graph of Fig.2 in the manuscript).

This setup allows simultaneous and quantitative acquisition of the two in-plane magnetization components during the hysteresis loop, i.e., the parallel ($M_{||}$) and transverse ($M_{\perp}$) magnetization components with respect to the applied magnetic field (see Fig. S1), for a given orientation, allowing full angular studies. It is able to perform automated angular-dependent dynamical magnetic measurements at RT, without readjusting the reflection plane or changing the Kerr geometry during the process. $\alpha_{\mathrm{H}} = 0°$ corresponds to the field direction parallel to the magnetic anisotropy easy axis (e.a.). The dynamic measurements were carried out by using triangular and sinusoidal magnetic fields from quasi-static (mHz) to 5 kHz and from 10 Hz to 150 kHz, respectively. A sinusoidal signal has only the fundamental frequency, without odd-harmonics, allowing to increase the frequency range without suffering shape distortions. Hysteresis curves were

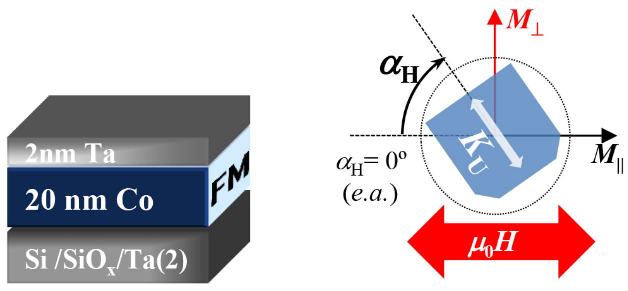

Fig. S1: ***Experimental.*** Scheme of sample structure (left) and experimental configuration (right), indicating the directions of the anisotropy K_{U}, sample angle (α_{H}), fixed external magnetic field ($\mu_0 H$), and magnetic sensitivity ($M_{||}$ and $M_{\perp}$).

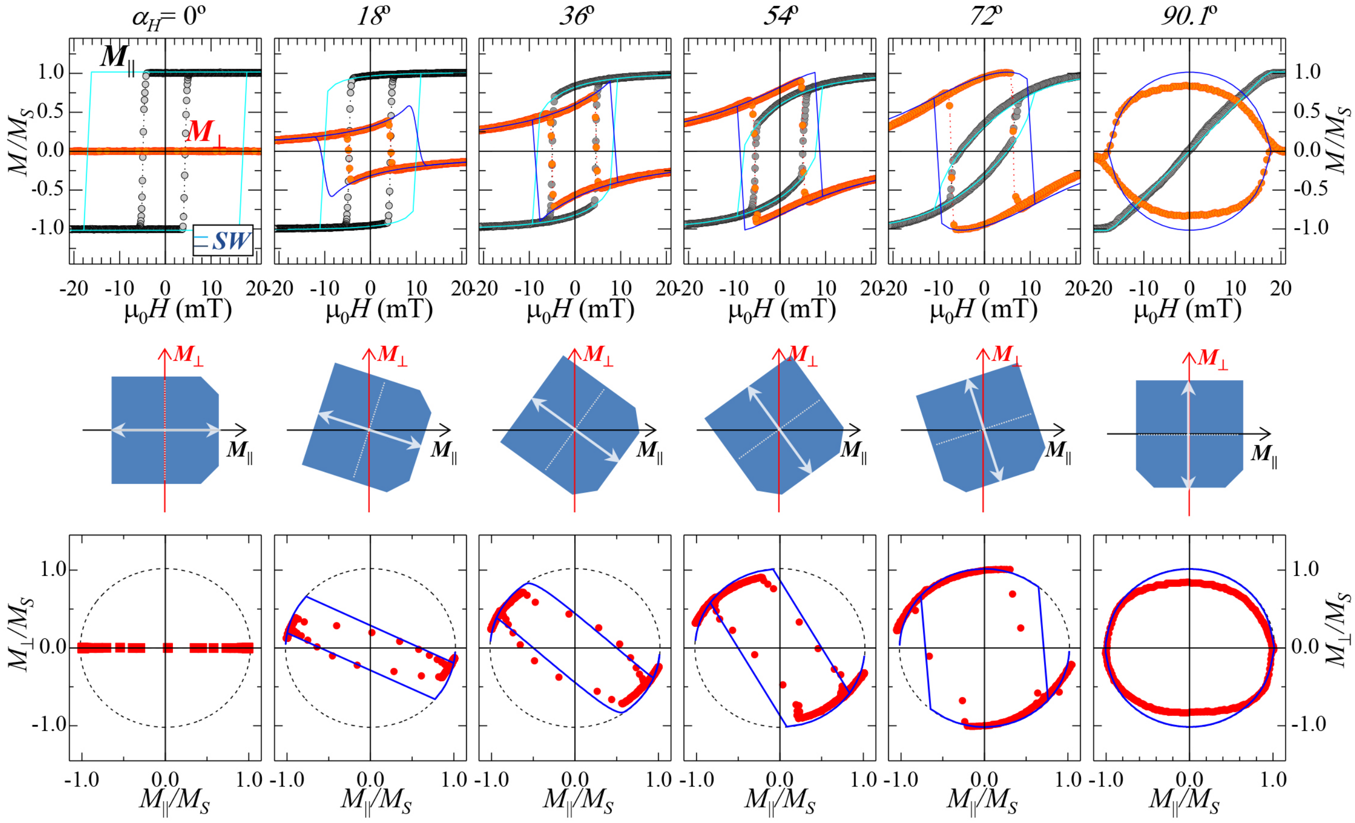

Fig. S2: ***Angular-dependent quasi-static hysteresis loops.*** Angular-dependent quasi-static hysteresis loops of a FM thin film with well-defined uniaxial magnetic anisotropy. The applied magnetic field angle α_{H} is indicated. (Top): standard $M-H$ representation. Symbols represent the parallel $M_{||}$ and transversal $M_{\perp}$ magnetization Kerr hysteresis loops acquired simultaneously. The descending (forward) and ascending (backward) field branches are depicted with filled and empty symbols, respectively. The solid lines correspond to the loops expected from SW model calculation. (Middle): experimental configuration scheme. Solid arrow ended and dashed straight lines indicate the position of the e.a. and h.a. directions, respectively. (Bottom): corresponding polar plot $M_{\perp}-M_{||}$ representation. Filled and open symbols have been used for the forward and the backward branch, respectively. The circle of radius unity depicted with a dashed line indicates the pure rotation of the magnetization vector. The solid lines correspond to the behavior expected from SW model.

obtained by averaging over several 10-1000 magnetization cycles to improve statistics. Two different set of experiments were performed. Detailed angular-dependent measurements over the whole angular range at representative dH/dt values (e.g., Fig.S1 and Fig.2), and detailed dynamic hysteresis measurements at representative α_{H} angles (e.g., Fig.2 and Fig.3). In both cases, the results are cross-checked for completeness.

QUASI-STATIC STUDY BY V-MOKE

Hysteresis and magnetization reversal

Representative in-plane resolved hysteresis loops acquired in quasi-static conditions at different α_H angles are shown in Fig. S2. The angles have been selected to show the rich variety of hysteresis and magnetization reversal processes that a single film with well-defined uniaxial magnetic anisotropy presents. Two different representations, standard $M-H$ (top graphs) and polar $M_{\perp}-M_{||}$ (bottom graphs) curves, have been used in order to identify relevant magnetic properties. In the middle, the sample orientation has been depicted for each case with the corresponding relative directions. Remarkably, a simple inspection of the in-plane resolved hysteresis loops provides direct information about the magnetization easy-axis (e.a.) and hard-axis (h.a.) directions, critical fields, domain wall angles and magnetization reversal processes.

At a first glance, vectorial (in-plane) resolved hysteresis loops display different magnetization reversal pathways in both magnetization components, which strongly depend on α_H, highlighting the importance of the simultaneous determination of both components provided by our setup. The different pathways are more evident for the $M_{\perp}(H)$ loop. In general, irreversible (sharp) transitions and/or fully reversible (smoother) transitions are observed in both $M_{||}(H)$ and $M_{\perp}(H)$ loops, corre-

sponding to nucleation of magnetic domains followed by domain wall propagation and rotation processes respectively. The relative weight of these two reversal mechanisms depends on α_{H}. The directional dependence originates from the symmetry breaking introduced by the magnetic anisotropy of the film.

At $\alpha_{\mathrm{H}} = 0°$ the parallel component presents a perfect squared shape hysteresis loop (top left graph of Fig. S2). $M_{||}$ does not change from the saturation (M_{S}) to the remanence ($M_{\|,\mathrm{R}}$), i.e., $M_{\|,\mathrm{R}}/M_{\mathrm{S}} \approx 1$, and there is only a sharp irreversible jump at the coercive field $\mu_0 H_{\mathrm{C}}$, in which the magnetization reverses. In turn the perpendicular component is negligible in the whole field swept, i.e., $M_{\perp}(H) \approx 0$. Both are expected behaviors of a magnetization easy-axis (e.a.) direction, in which the magnetization reversal takes place via nucleation and subsequent propagation of magnetic domains oriented parallel to the field direction.

For $\alpha_{\mathrm{H}} \neq 0°$, clear $M_{\perp}(H)$ loops with both reversible and irreversible transitions are found, in correspondence to the $M_{||}(H)$ loops, as shown in the central graphs of Fig. S2 (from 18° to 72°). In addition, larger $M_{\perp}$ signals are found as α_{H} increases, which indicates that the rotation processes (reversible transitions) becomes more relevant and that the magnetic domains nucleated during the irreversible transition are not oriented parallel to the field direction but to the e.a. direction, in accordance with the expected reversal features of well-defined uniaxial magnetic anisotropy systems. According to this, at $\alpha_{\mathrm{H}} = 90°$ the $M_{||}(H)$ loop shows an almost linear and reversible behavior of the magnetization, $M_{||,\mathrm{R}}/M_{\mathrm{S}} \approx 0$, and $\mu_0 H_{\mathrm{C}} \approx 0$ mT (see right-most graph of Fig. S2), typical of an uniaxial magnetic anisotropy hard-axis (h.a.) direction. Notice that this selected angle is not exactly $\alpha_{\mathrm{H}} = 90°$. If that were the case, a negligible signal would be found in the corresponding $M_{\perp}(H)$ loop because of a perfect alignment of the external field with the h.a. direction in conjunction with the acquisition procedure. To be more exact, a vanishing perpendicular component is the consequence of averaging during many successive iterations, where for each one of them, the magnetization would rotate alternatively along the positive and negative values of $M_{\perp}$. A little misalignment (notice that is just 0.1° in right-most graph of Fig. S2) ends up with a rather circular $M_{\perp}(H)$ loop since the misalignment promotes a selection of one of the sense of the rotation, alternated for each of the two semi-cycles of the loop.

The quantitative information obtained from our v-MOKE setup allows the visualization of the in-plane trajectory of the magnetization vector during reversal (see bottom graphs of Fig. S2). In this polar plot representation, the data lying on the circle of radius unity, depicted with a dashed line, represent rotation processes. Every time the data is off this circle, magnetic domains are present. The specific mechanisms of the magnetization reversal are easily to detect with this plots. In all the cases, except for e.a., as the field is decreased from the maximum field, the magnetization vector rotates reversibly along the circle of radius unity. The rotation continues beyond zero field with the opposite field sense until an irreversible process is initiated, as indicated by the departure of the magnetization vector from that circle. Notice that both departure and return points are closed to e.a. direction, which accounts for irreversible process due to nucleation of magnetic domains oriented along the e.a. directions and subsequent domain wall propagation. The return point is found c.a. 180° away from departure point, depending on α_H. Note that the magnetization vector is far from being saturated along the field direction even for the largest field used, except for $\alpha_H = 0°$.

Therefore, the magnetization reversal proceeds by reversible magnetization processes, starting at saturation magnetization, all the way up to the closest e.a. direction, and from there it continues with a subsequent irreversible transition, which is related to nucleation of antiparallel magnetic domains and subsequent propagation of 180° domain walls. In addition, the magnetic domains are not oriented necessarily parallel to the field direction but rather to the e.a. direction. In order to have quantitative analysis, relevant magnetic properties associated with the reversible and irreversible processes are discussed in the following.

Relevant magnetic parameters, such as remanences and critical fields can be readily obtained as a function of α_H from the hysteresis loops, as indicates in Fig. S3. In both cases, the parameters are obtained after averaging the corresponding values of the forward and backward field branches at zero field and during reversal transitions

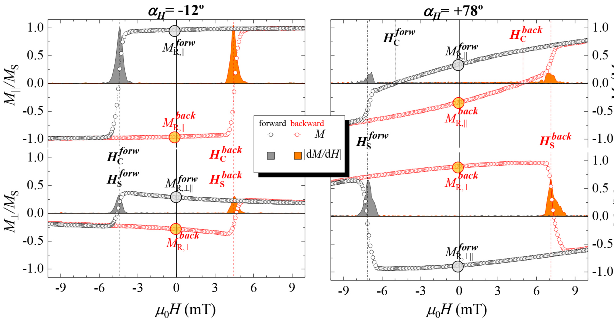

Fig. S3: ***Hysteresis and magnetic parameters.*** Vectorial-resolved hysteresis loops acquired at two representative α_H angles to illustrate the procedure to extract the magnetic parameters. The symbols are the experimental $M_{||}(H)$ and $M_{\perp}(H)$ loops and the solid lines with filled colored areas are their corresponding derivative curves. The descending (forward) and ascending (backward) field branches are depicted with different colors. The remanences $M_{\mathrm{R},||}$ and $M_{\mathrm{R},\perp}$ are indicated. The vertical dotted and dashed lines indicate the coercivity H_{C} and the switching field H_{S}, respectively.

for the remanences and the critical fields, respectively. In this sense, the two in-plane remanence values reads: $M_{\mathrm{R},\parallel} = \frac{1}{2}(M_{\mathrm{R},\parallel}^{\mathrm{forw}} - M_{\mathrm{R},\parallel}^{\mathrm{forw}})$, $M_{\mathrm{R},\perp} = \frac{1}{2}(M_{\mathrm{R},\perp}^{\mathrm{forw}} - M_{\mathrm{R},\perp}^{\mathrm{forw}})$. Notice that $M_{\mathrm{R},\parallel}$ is always positive, whereas $M_{\mathrm{R},\perp}$ can be either positive (e.g., Fig. S3(a)) or negative (e.g., Fig. S3(b)), so there must be an angle where it vanishes. That angle is used to identify the characteristic magnetization easy-axis and hard-axis directions with great accuracy (see Fig.4a). In turn, the critical fields reads: $H_{\mathrm{C}} = \frac{1}{2}(H_{\mathrm{C}}^{\mathrm{back}} - H_{\mathrm{C}}^{\mathrm{forw}})$; $H_{\mathrm{S}} = \frac{1}{2}(H_{\mathrm{S}}^{\mathrm{back}} - H_{\mathrm{S}}^{\mathrm{forw}})$. The coercive field H_{C} refers to the magnetic field required to reduce the magnetization to zero, i.e., $M_{\parallel}(H_{\mathrm{C}}) = 0$, whereas the switching field H_{S} is the magnetic field where the irreversible transition takes place. In analogy, H_{S} can be derived from the magnetization curve of the transverse component, i.e., $M_{\perp}(H_{\mathrm{S}}) = 0$, or more accurately from its derivative curve (shadow area in Fig. S3) looking for the field where its maximum is located (indicated with vertical dashed lines in Fig. S3). Notice that there is an angular range around the e.a. where both coercivity and switching field coincide (e.g., Fig. S3(a)), and in particular for $\alpha_H < \pm 70°$). Within (out of) this angular range, coercivity and switching field have the same (different) meaning, as discussed below.

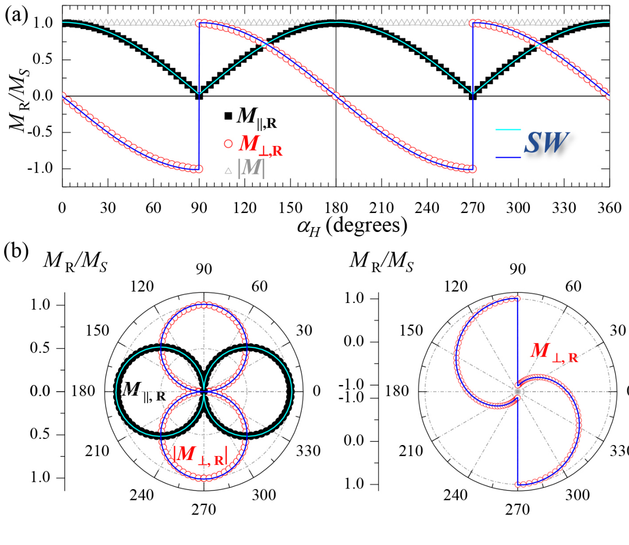

Fig. S4: ***Remanence and magnetic symmetry.*** (a) Angular dependence of the normalized remanent magnetization components of a well-defined uniaxial magnetic anisotropy system in quasi-static conditions. (b) Corresponding polar plot representations. The values of $M_{\mathrm{R},\parallel}$ (filled square symbols) and $M_{\mathrm{R},\perp}$ (open circle symbols) have been extracted from the in-plane resolved hysteresis loops, as the ones shown in Fig. S2. Solid lines are derived from the SW model.

Remanence and magnetic symmetry

The simplest information that can be extracted from the angular evolution, is the anisotropy directions, determined by the magnetic symmetry of the system. This can be done easily by plotting the normalized remanence values of the two in-plane magnetization components as a function of α_H (see Fig. S4). Both magnetization components display a pronounced oscillation with periodicity of 180°. The parallel component follows a $|\cos 2\alpha_H|$ law dependence, the perpendicular component changes the sign when a characteristic e.a. or h.a. direction is crossed, and both components are complementary, i.e., $M_{\mathrm{S}}^2 = (M_{\mathrm{R},\parallel}^2 + M_{\mathrm{R},\perp}^2)$. The polar plots of $M_{\mathrm{R},\parallel}$ and $M_{\perp,\mathrm{R}}$ display characteristic *"two-lobes"* shapes rotated 90° (see Fig. S4(b)). This originates from the two-fold magnetic symmetry induced by the field applied during growth. All these features confirm a well-defined uniaxial magnetic anisotropy behavior of the film, where the anisotropy axis was field-induced as already explained.

Irreversible transitions and relevant fields

The more relevant transition fields are the coercivity ($\mu_0 H_{\mathrm{C}}$) and the switching field ($\mu_0 H_{\mathrm{S}}$), which are related to the field required to compensate the magnetization along the field direction and to the effective anisotropy of the system, respectively. Coercivity describes the stability of the remanent state and gives rise to the classification of magnets into hard magnetic materials (permanent magnets), semihard materials (storage media) and soft magnetic materials (core applications). The switching field determines the field needed to control the stable magnetization direction in any magnetic system, crucial in both permanent magnet and spintronic applications. Both can easily be determined from the $M - H$ curves by looking for the applied field values at which $M_{\mathrm{R},\parallel}$ and $M_{\mathrm{R},\perp}$ crosses zero, respectively. Notice that the coercive field does not always indicates the field value at which the irreversible transition takes place, while the switching field does. The sharp irreversible transitions are associated with the nucleation field and results in an abrupt change in the $M - H$ loop, i.e., in a large peak in the corresponding numerical derivative curves (see Fig. S3). Notice that the irreversible hysteresis jumps found in both components, and their corresponding derivative curves, are not necessarily of the same height. Close to the e.a. direction the $M_{\parallel}(H)$ loops present significantly larger irreversible jumps, and higher maximum values of the corresponding derivative curves, in comparison with the ones found in the $M_{\perp}(H)$ loops, whereas approaching the h.a. direction the difference exchanges, as the right graphs of Fig. S3 shows. In this case, the angular evolution of switching field is more precise to determine from the $M_{\perp}(H)$ loops.

Fig. S5 displays the angular dependence of the relevant fields extracted from $M-H$ loops. In general, the two-fold symmetry of the magnetic properties is also found in the experimental data of the angular dependence of both $\mu_0 H_C$ and $\mu_0 H_S$, as revealed by the 180° periodicity presented in both cases (Fig. S5(a)). The coercive field presents a roughly constant value around the e.a. direction, i.e., $\mu_0 H_C(0°) = 4.5$ mT, increasing slightly until $\alpha_H \approx \pm 75°$. The switching field presents an identical angular evolution in this angular range. Above this angle, $\mu_0 H_C$ starts to decrease, vanishing at the h.a. direction, i.e., $\mu_0 H_S(90°) = 0$ mT. In contrast, $\mu_0 H_S$ presents a larger rising evolution as the angle approaches to the h.a. direction. The switching field at the h.a. direction is the effective anisotropy field, i.e., $\mu_0 H_S(90°) = \mu_0 H_K = 17$ mT. Notice that there is a factor of nearly four between the coercive field at the e.a. and the anisotropy field.

In order to understand the angular evolution of the relevant fields, we have compared the experimental results in quasi-static conditions with the expected ones from two, in principle, opposite models, which assumes rotation and single domain switching (SW model [2]) and rotation and nucleation of magnetic domains and subsequent propagation of domain walls pinned at defects (pinning-model [3]). There are two angular ranges where each model reproduces the experimental data. The SW model assumes a single particle behavior, i.e., reversal by switching (irreversible process) and/or rotation (reversible) of the whole magnetization, without taking into account any other irreversible magnetization reversal process like nucleation and propagation of magnetic domains. The latter is, in fact, energetically much more favorable in extended systems, in which defects of any kind—structural or morphological—play the main role to activate irreversible magnetic domain nucleation processes at magnetic fields that are significantly lower than expected by the SW model. In particular, even in epitaxial thin films, low coordination sites such as sample edges and topographic roughness are unavoidable. This defects will act as pinning centers for the created magnetic domain walls, and the reversal subsequently continues via domain wall propagation, as the field pressure allows the wall to unpin (overcoming its local energy barrier), just to reach other (higher energy barrier) pinning centers, and so on until all the reversal is completed. In this case, a simple model of pinned 180° magnetic domain walls (pinning model) predicts a $1/|\cos\alpha_{\rm H}|$ law for the angular dependence of the reversal field, which has been already observed in both perpendicular [4] and in-plane [5] anisotropy systems.

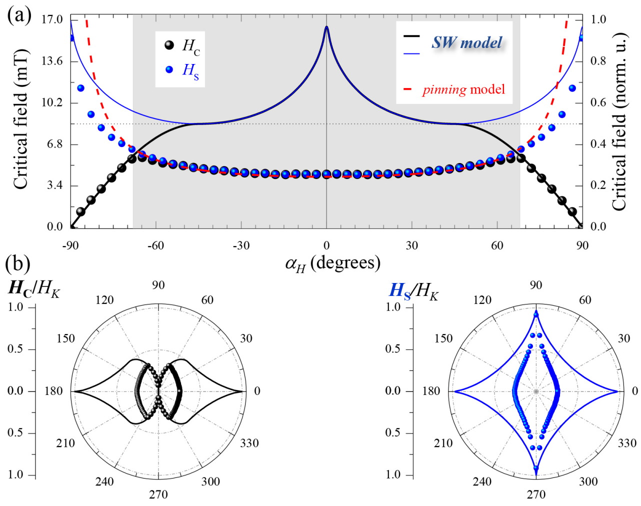

Fig. S5: ***Coercivity and switching fields.*** (a) Angular dependence of the critical fields for a well-defined uniaxial magnetic anisotropy film in quasi-static conditions. H_C (solid symbols) and H_S (open symbols) are the experimental coercivity and switching field values, respectively (see Fig. S2). Solid and dashed lines are the expected behaviors from the SW model and the pinning model, respectively (see text). (b) Corresponding polar plot representations. Notice that the experimental data are reproduced with the two models in different angular regions where the irreversible (pinning) and reversible (SW) processes are more relevant during magnetization reversal, and the irreversibilities are associated with a more propagative and nucleative regime, respectively.

In a wide angular region around the e.a. direction, i.e., $|\alpha_{\rm H}| < 70°$, both fields are similar and follows a $1/|\cos\alpha_{\rm H}|$ law (discontinuous line in Fig. S5(b)), accordingly to the domain pinning model prediction. Thus, nucleation and subsequent propagation of pinned magnetic domains is the relevant process during the irreversible transitions. Close to the h.a. direction, i.e., $|\alpha_{\rm H}| > 70°$, the pinning model cannot reproduce the experimental data, and the predicted evolution tends to separate more and more from experimental values. On the other hand, SW model, which does not reproduce well the experimental results below 70° becomes closer and closer as h.a. is reached, reproducing quite satisfactory the results at the exact h.a. For instance, the coercive field (switching field) decreases (increases) to zero (up to the anisotropy field) as approaching the h.a. direction, as predicted by the SW model (solid lines in Fig. S5). This indicates that close to the h.a. direction the magnetization reversal is mainly governed by rotation processes. Note that the SW model only reproduces their angular evolution around the h.a. directions, where reversible processes are the relevant mechanism during reversal (clear area in Fig. S5). It fails around the e.a. directions, where irreversible (nucleation and propagation of magnetic domains) processes dominate, as described above.

In summary, the quasi-static magnetic properties of a well-defined uniaxial magnetic anisotropy system have been disentangled by means of a detailed angular dependence vectorial-resolved magnetization reversal study. In general, both reversible and irreversible transitions ta-

kes place during reversal, and their relevance depends strongly on $\alpha_{\rm H}$. The reversible transitions correspond to magnetization rotation processes whereas the irreversible ones are related to nucleation of magnetic domains aligned with respect to the anisotropy axis and a subsequent domain wall propagation. The data are discussed in the framework of both domain wall pinning and macrospin SW reversal models for different angular ranges. In particular, the pinning and SW models reproduce the experimental data close to the e.a. and h.a., respectively.

* Electronic address: julio.camarero@uam.es.

[1] E. Jiménez, N. Mikuszeit, J. L. F. Cuñado, P. Perna, J. Pedrosa, D. Maccariello, C. Rodrigo, M. A. Niño, A. Bollero, J. Camarero, and R. Miranda, Rev. Sci. Instrum. **85**, 053904 (2014); Rev. Sci. Instrum. **86**, 046109 (2015).

[2] E. C. Stoner, E. P.Wohlfarth, E. P. (1948). Philos. Trans. R. Soc. London, Ser. A **240**, 599 (1948); IEEE Trans. Magn. **27**, 3475 (1991).

[3] S. Chikazumi, *Physics of Ferromagnetism*, Oxford Science Publications, New York (1997), pp. 118-119, pp. 491.

[4] D. Givord, P. Tanaud, and T. Viadieu, J. Magn. Magn. Mater. **72**, 247 (1988)

[5] P. Perna, C. Rodrigo, E. Jiménez, N. Mikuszeit, F. J. Teran, L. Méchin, J. Camarero, and R. Miranda, J. Appl. Phys. **110**, 1391 (2011).
